# Editorial: Recent advances in the prevention, diagnosis and treatment of Chagas disease

**DOI:** 10.3389/fmicb.2024.1537105

**Published:** 2024-12-20

**Authors:** Giovanna Frechiani, Adalberto Vieyra, Claudia F. Dick

**Affiliations:** ^1^Graduate Program in Translational Biomedicine/BIOTRANS, Grande Rio University/UNIGRANRIO, Duque de Caxias, Brazil; ^2^National Center for Structural Biology and Bioimaging, Carlos Chagas Filho Institute of Biophysics, Federal University of Rio de Janeiro, Rio de Janeiro, Brazil; ^3^National Center for Structural Biology and Bioimaging/CENABIO, Federal University of Rio de Janeiro, Rio de Janeiro, Brazil

**Keywords:** trypanosomatids, new therapeutic strategies, Chagas disease diagnosis, Chagas disease, *Trypanosoma cruzi*

## Introduction

Chagas disease (CD), also known as American trypanosomiasis, is caused by the parasite *Trypanosoma cruzi*. It is prevalent in areas belonging to South and Central America, but in the global scenario, the disease ranks third among parasitic diseases (World Health Organization, 2023). Currently, it is estimated that around 10 million people are infected worldwide, especially in Latin America, where the disease is endemic and is the leading cause of non-ischemic heart disease (Pan American Health Organization, 2023). *T. cruzi* is mainly transmitted by kissing bugs hematophagous insects belonging to the subfamily Triatominae. Thus, CD stands out as a public health problem in Latin America and other continents, mainly in North America and Europe. This spread is due to the immigration of infected individuals and the expansion of the insect vector in border areas, in addition to the failure to control clinical procedures such as blood transfusions and organ transplants (Nunes et al., 2018). Another form of transmission is the congenital form. Congenital infection with *T. cruzi* has become the primary mode of CD transmission in non-endemic countries where pregnant women are not regularly screened for the infection (Antinori et al., 2017). At the maternal-fetal interface, a complex interaction takes place between the parasite and host cells when bloodstream trypomastigotes engage with the syncytiotrophoblasts (SYNs) (Blaszkowska and Goralska, 2014; Silberstein et al.). RNA sequencing, complete transcriptome analysis, and 3D-cultured placental trophoblasts were used to describe the SYN response to *T. cruzi* and validate their crucial function as immunological sensors of parasite infection. Knowledge of the placenta's immunological environment, particularly the role of SYNs and the regulation of innate immunity, may aid in creating novel treatments to lower the risk of congenital CD. Both the acute and chronic phases of maternal infection can result in this kind of infection; moreover, pregnant women with greater parasitemia have higher transmission rates (Silberstein et al.).

## Diagnosis

The diagnosis of CD is fraught with significant challenges, primarily due to cross-reactivity in serological tests with other pathogens, such as *Leishmania* spp. and *Trypanosoma rangeli* (Ascanio et al.). This Research Topic is particularly problematic in situations like blood or organ donation, where inaccurate results can compromise patient safety. In the chronic phase, the use of multiple assays becomes necessary to confirm the diagnosis, tests that encompass a variety of approaches, each with its specific characteristics (World Health Organization, 2023). In response to this complex scenario, numerous researchers have intensified their efforts to develop and test new antigens, such as recombinant proteins. These recombinant antigens offer several advantages, including increased sensitivity and specificity. Despite being in smaller quantities, recombinant multiepitope proteins have also shown improved diagnostic performance, significantly enhancing diagnostic accuracy and providing a reassuring outlook for the future of CD diagnosis (Resende et al.). Among the serological tests, ELISA (Enzyme-Linked Immunosorbent Assay) is widely used to detect antibodies against *T. cruzi* and is considered one of the most sensitive and specific methods available. Another option is the RDT (Rapid Diagnostic Test), which provides results quickly, making it especially useful in areas with limited resources. In addition to molecular methods, where PCR (Polymerase Chain Reaction) detects *T. cruzi* DNA in blood samples, RDT is especially effective in acute cases and patients with high parasitemia (Ascanio et al.).

## Treatment

Despite the availability of some drugs in the clinic, these therapies have significant limitations. The toxicity associated with available medications, especially nifurtimox and benznidazole, often limits treatment adherence, given the adverse effects of continued use (Ferri et al.; Gonzaga et al.). Although such treatment shows results in the acute phase of the disease, its effectiveness in the chronic phase is considered limited. In addition to controlling parasitemia, no molecules are available in the industry that act to reverse the damage already caused by cardiac or hepatic complications in advanced stages (Altcheh et al., 2021). Many studies attempt to reduce this damage caused by the current treatment available for the disease. Some studies look for molecules acquired from natural compounds, such as those found in hawthorn extract (*Crataegus oxyacantha*), which can inhibit the Epac-Rap1b pathway, reducing invasion levels comparable to no treatment (Ferri et al.). Among the natural compounds found easily in different environments, both in the gut of the insect and in the bloodstream of the mammalian host, others are acquired naturally from the diet (Dick et al., 2020). The parasite finds competition for these nutrients in the mammalian host's bloodstream environment and within the insect vector's gut. The intestinal microbiota of the vector competes with *T. cruzi* for essential nutrients, such as sugars, amino acids, and Fe, all crucial elements for the energy metabolism of the protozoan (Villacís et al.). This competition can limit the availability of Fe in the environment, impairing processes such as cellular respiration, which may influence the growth of the parasite, compromising the glycolytic and oxidative metabolism of the protozoan (Orantes et al., 2018).

As previously presented, the urgent need for new therapeutic targets for CD is underscored by the limited therapeutic arsenal. This pressing need necessitates the development of more effective and less toxic approaches that consider different disease mechanisms. Therefore, the proposed new targets offer promising approaches for their use alone or in combination with existing therapies.
